# Dark Times: The Role of Negative Reinforcement in Methamphetamine Addiction

**DOI:** 10.3389/fpsyt.2020.00114

**Published:** 2020-03-17

**Authors:** April C. May, Robin L. Aupperle, Jennifer L. Stewart

**Affiliations:** ^1^Joint Doctoral Program in Clinical Psychology, San Diego State University and University of California, San Diego, San Diego, CA, United States; ^2^Laureate Institute for Brain Research, Tulsa, OK, United States; ^3^Department of Community Medicine, University of Tulsa, Tulsa, OK, United States

**Keywords:** methamphetamine, negative reinforcement, emotion regulation, depression, anxiety, substance use disorder, neuroimaging, treatment

## Abstract

Methamphetamine use is associated with substantial adverse outcomes including poor mental and physical health, financial difficulties, and societal costs. Despite deleterious long-term consequences associated with methamphetamine, many people use drugs for short-term reduction of unpleasant physical or emotional sensations. By removing these aversive states, drug use behaviors are negatively reinforced. Abstinence from methamphetamine can then result in a return to previous aversive emotional states linked to withdrawal and craving, often contributing to an increased likelihood for relapse. This negative reinforcement cycle is hypothesized to be a motivating and maintaining factor for addiction. Thus, this review highlights the current evidence for negative reinforcement mechanisms in methamphetamine use disorder by integrating studies of subjective experience, behavior, functional magnetic resonance imaging, positron emission tomography, and event-related potentials and examining the efficacy of treatments targeting aspects of negative reinforcement. Overall, the literature demonstrates that individuals who use methamphetamine have diminished cognitive control and process emotions, loss of reward, and interoceptive information differently than non-using individuals. These differences are reflected in behavioral and subjective experiments as well as brain-based experiments which report significant differences in various frontal regions, insula, anterior cingulate cortex, and striatum. Together, the results suggest methamphetamine users have an altered experience of negative outcomes, difficulties employing effective emotion regulation, and difficulty engaging in adaptive or goal-directed decision-making. Suggestions for future research to improve our understanding of how negative reinforcement contributes to methamphetamine addiction and to develop effective interventions are provided.

## The Role of Negative Reinforcement in Methamphetamine Addiction

Methamphetamine is a commonly abused illicit substance due to its stimulating and euphoriant effects. However, its use is also associated with many consequences at the individual and societal level. For the individual, methamphetamine use can result in significant physical and mental health effects, including but not limited to cardiovascular/cerebrovascular dysfunction and mortality, depression, anxiety, cognitive deficits, psychosis, violence, and suicide (1, 2). In fact, suicide has been estimated to account for 18.2% of all methamphetamine-related deaths (3) and approximately 1/3 of adults addicted to methamphetamine report having attempted suicide one or more times (4). Additional public health concerns include high rates of crime and a significant burden on the health care system due to the deleterious physical effects of methamphetamine. According to the most recent National Survey on Drug Use and Health (5), methamphetamine use in the United States has increased since 2017, with approximately 1 million individuals using in the past month and over 1.8 million using in the past year. Given the severe consequences and increasing prevalence of methamphetamine use, it is important to understand reinforcing mechanisms that maintain and escalate symptoms of methamphetamine use disorder.

Drug use is commonly understood as providing immediate short-term reward. This acute positive effect of the substance (e.g., euphoria and/or high) can be seen behaviorally and within brain regions implicated in reward, including medial orbitofrontal cortex (OFC), rostral anterior cingulate cortex (ACC), and ventral striatum, in frequent users as well as substance-naïve individuals (6). When these positive feeling states outweigh the negative consequences and perpetuate use, drug-seeking behavior is said to be positively reinforced. However, methamphetamine use may also be reinforced by alleviating or removing uncomfortable or aversive states within the body. This principle, known as negative reinforcement, suggests that individuals continue to use drugs, despite negative consequences, because it alleviates uncomfortable states or sensations such as those associated with negative mood states, tension, arousal, craving, or withdrawal. For some individuals, these uncomfortable states and situations develop as a symptom of withdrawal following periods of prolonged use. For others, even initial use can be used as a maladaptive coping mechanism to alleviate aversive states that existed prior to drug use such as depression, anxiety, or reduced responsivity to reward.

**Figure 1:**
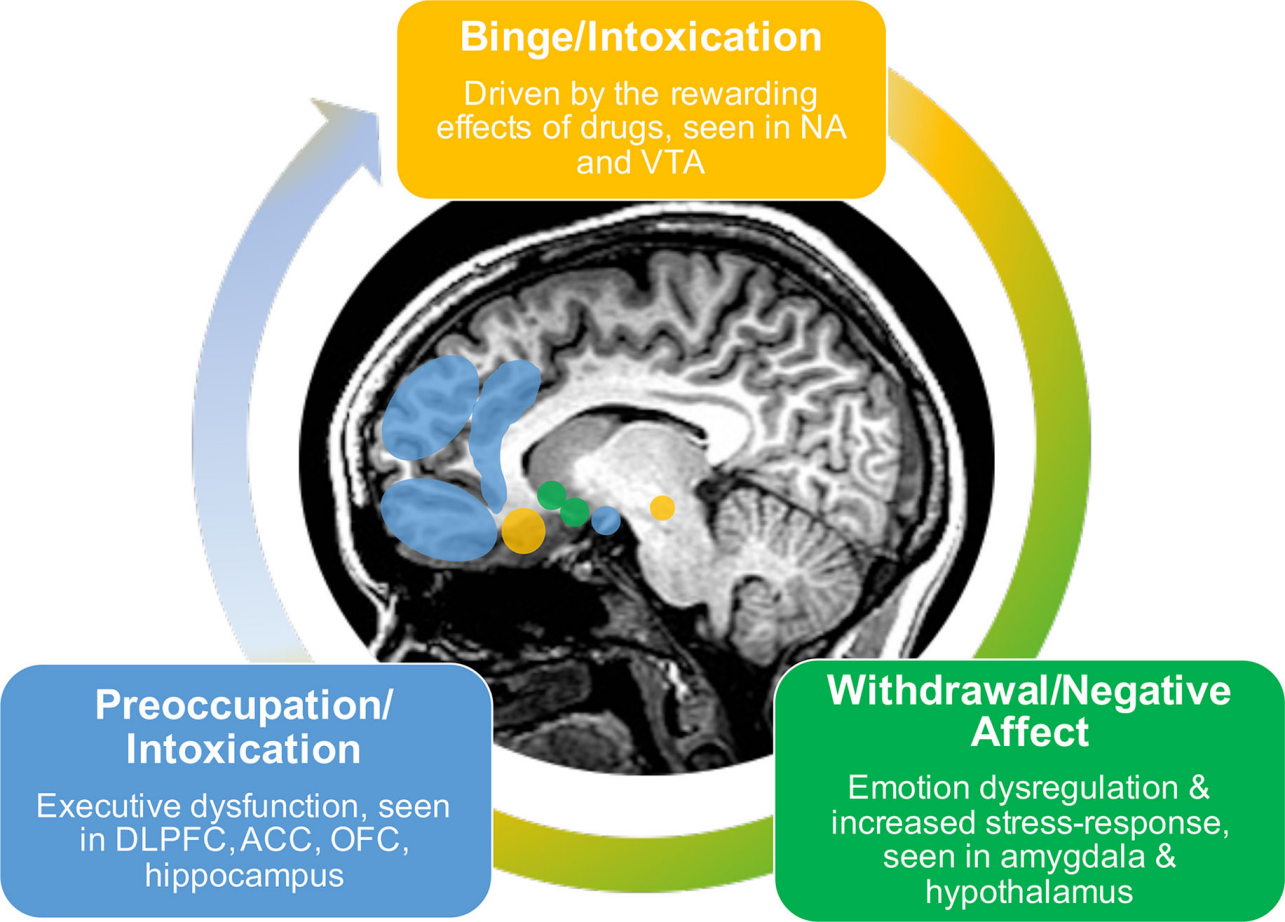
Three-stage model of addiction.

A recent conceptualization describes addiction as a three-stage cycle of binge/intoxication, withdrawal/negative affect, and preoccupation/anticipation marked by varying dysfunction among motivation, reward, stress, and executive function systems (**Figure 1**) (7–9). The initial state of binge/intoxication is driven by the rewarding effects of drugs, in which an increased incentive salience is attributed to the drug and new drug-seeking habits develop. During the withdrawal/negative affect stage, the individual experiences increases in negative emotional states and an overall increased stress-response. The third stage of preoccupation/anticipation consists of increased drug-craving and deficits in executive functioning. These three stages are hypothesized to feed into one another, increase in intensity over time, and ultimately result in addiction (7). Addiction can therefore be thought of as an evolving process in which initial use is positively reinforced by the rewarding effects. However, with sustained use it becomes negatively reinforced as it relieves negative states including irritability, physical pain, emotional symptoms, such as depression and anxiety, and blunted responsivity to natural rewards [e.g., pleasant social interactions, food, water, monetary gain; Koob, (7)]. Negative reinforcement is therefore hypothesized to play a key role in the development and maintenance of addiction.

This conceptualization of addiction as a three-stage cycle can be demonstrated through findings from animal studies. During sessions of drug self-administration, animals will titrate their intake based on availability; as drug availability increases, animals significantly increase their self-administration of various drugs including methamphetamine, cocaine, nicotine, heroin, and alcohol (10–14). With continued administration, the drug’s incentive salience increases and new motivations to seek the drug develop, reflecting the initial binge/intoxication stage of addiction. With increased drug-intake, reward thresholds also increase, resulting in reduced responsivity to natural rewards (9). This increase in reward threshold correlates with amount of drug intake and does not return to baseline after cessation of the drug administration session (15). With protracted abstinence, animals demonstrate symptoms of withdrawal, corresponding to the withdrawal/negative affect stage of addiction. These symptoms include negative emotional states as demonstrated by anxiety-like responses on behavioral tests (16) such as conditioned place aversion, wherein the animal avoids a place previously paired with an aversive state (17). Over time, animals with increased access to drugs of abuse demonstrate working memory impairments, as well as changes in neuronal density and functional connectivity of various frontal regions (e.g., prefrontal cortex, PFC; OFC), thereby contributing to a loss of control resulting in compulsive drug use, and ultimately progressing to a state of addiction (18, 19).

While our understanding of negative reinforcement in addiction has grown in recent years, the extent to which it plays a role in perpetuating addiction in humans is still not well established. Therefore, this review consists of two main aims: (1) to evaluate the evidence for negative reinforcement in methamphetamine addiction; and (2) to examine how treating negative affective symptoms impacts substance use outcomes related to abstinence and well-being, given the need for effective interventions for methamphetamine addiction. Although negative reinforcement is believed to play a role in addiction more generally, the present review focuses solely on methamphetamine given the recent resurgence of use and use-related problems. Data from the Center for Disease Control show that overdose deaths related to methamphetamine use tripled from 2011 to 2016 (20), highlighting the need for effective prevention and intervention options. Additionally, the role of negative reinforcement has commonly been examined within the context of other drugs of abuse [e.g., opioids; Koob, (21)] but less work has been done to examine these processes in relation to methamphetamine use. Therefore, while the results reported in this review reflect the role of negative reinforcement in methamphetamine specifically, these findings may be used as a framework for understanding its role in substance use at large.

A literature search was conducted in the PubMed database using the search terms listed in **Table 1**. The same search was then conducted using Google Scholar. Any additional articles identified through Google Scholar were then accessed via PubMed to ensure they met eligibility criteria. To be included, studies were required to examine some component of negative reinforcement among adult methamphetamine users. Samples were required to consist of individuals with either a primary diagnosis of methamphetamine use disorder (MUD) as defined by endorsement of 2+ diagnostic criteria (22), methamphetamine dependence based on endorsement of 3+ diagnostic criteria (23), or methamphetamine abuse (MA) associated with endorsement of 1 symptom (23). Negative reinforcement could be examined within the context of negative emotions/affect, withdrawal, craving, losses, pain, rejection, and/or stress. The article selection process is detailed in **Table 1**.

**Table 1:** Search terms and article selection.

	Key words
**Drugs**	Methamphetamine, amphetamine, stimulant, dependence, use disorder, addiction, craving, withdrawal
**Brain**	Magnetic resonance imaging, fMRI, MRI, brain stimulation, repetitive, magnetic, event-related potential, positron emission tomography
**Negative reinforcement**	Depression, anxiety, (negative) affect, loss/es, (negative) emotion, stress, sad, angry, fearful, distress, pain, nociception, rejection
**Modality**	Human
	**Journal articles**
**# Evaluated**	190
**# Included**	Self-report/behavioral = 21; fMRI = 10; sMRI = 1; ERP = 1; PET = 6; treatment = 25
**Reasons for exclusion**	Review papers (*n* = 23); did not examine negative reinforcement variables (*n* = 67); not MUD focused (*n* = 25); case study (*n* = 3); acceptability/feasibility study (*n* = 4); adolescents (*n* = 2); rats (*n* = 2)

fMRI, functional magnetic resonance imaging; MRI, magnetic resonance imaging; ERP, event-related potential; PET, positron emission tomography; MUD, methamphetamine use disorder.

In the sections below, we review the evidence on the role of negative reinforcement in methamphetamine addiction based on self-report and behavioral data. We then describe functional and structural magnetic resonance imaging (fMRI; sMRI), event-related potential (ERP), and positron emission tomography (PET) studies aiming to provide insight into the neural mechanisms related to negative reinforcement in methamphetamine addiction. The details of the studies reviewed in these sections can be found in **Table 2**. Lastly, treatments for MUD that specifically address negative reinforcement mechanisms are evaluated (see **Table 3**), and implications for future interventions and research avenues are discussed.

**Table 2:** Subjective, behavioral, physiological, and brain-based findings of negative reinforcement in methamphetamine users.

Author (first author, year)	Meth Group (N)	Comparison Group (N)	Abstinence Duration (Days)	Meth Chronicity [M(SD)]	Comorbid Diagnoses	Gender Examined?	Methods	NR Variables	Results ↑↓
***Self-Report findings***									
**Newton et al**. (24)	73 non-treatment seeking, current users	None	N/A	10.6(8.2) yrs	No Axis I psychiatric disorders, no dependence on drugs other than MA or nicotine	No	Self-report questionnaires	Reasons for taking drugs	23% of respondents reported negative reinforcement reasons for substance use
**Tayyebi et al**. (25)	40	40 narcotics users, 40 CTL	N/R	N/R	N/R	No	Self-report questionnaires	Self-regulation, affective control	MA< narcotics users & CTL: self-regulation and affective control
***Behavioral and Physiological Findings***									
**Chen et al**. (26)	60	30	4.85(1.12) months	33.12(24.99) months	N/R	Yes; no sig. diff. found	Startle response measured by skin conductance	Self-report emotional response, startle response, skin conductance	MA: ↑ emotional response to anger-eliciting videos, ↓ emotional response to joy-eliciting videos
**Henry et al**. (27)	12	12	5.9(1.41) months	3.9(2.16) yrs	N/R	No	Facial affect recognition task	Ability to identify emotions	MA: ↓ facial affect recognition
**Kim et al**. (28)	28	27	19.46(7.86) days	13.93(7.76) yrs	N/R	No	Facial affect recognition task	Ability to identify emotions	MA: ↓ facial affect recognition
**Zhong et al**. (29)	54	58	44.85(20.65) days	4.14(3.42) yrs	75.9% of MA reported history of psychiatric symptoms	No	Baseline, 3-and 6-months abstinent	Ability to identify emotions	MA: ↓ social emotional cognition at baseline but improvement after 6-months abstinence
***Sex-Specific Findings***									
**Chen et al**. (30)	30 females	30 females	8.68(3.64) months	35.23(22.41) months	N/R	No; females only	Cross-sectional	Startle response & self-reported arousal & valence of emotional music stimuli;	Startle, MA<CTL for fearful stimuli; Self-report arousal: MA<CTL for fearful and happy stimuli; Self-report valence: MA>CTL for fearful stimuli
**Hartwell et al**. (31)	203	None	1.6(3.6) days	N/R	5.4% current MDD	Yes	One-time self-report assessment	Dep. & Anx. symptoms, craving	Within males only: Positive corr. b/w Dep. symptoms & craving, Positive corr. b/w Anx. symptoms and craving
**Maxwell et al**. (32)	222	None	N/R	N/R	N/R	Yes	One-time self-report assessment	Motivations for MA use	Female > male: using MA to “not feel depressed”
**Mehrjerdi et al**. (33)	80	80	N/R	5(6.1) yrs of dependence, years of use not reported	N/R	No; females only	Cross-sectional	Coping strategies	MA<CTL: seeking social support, cognitive evaluation, problem-solving; MA>CTL: emotion control, physical control
**Shen et al**. (34)	113 females	None	8.7(4.8) months of detoxification	2.0(1.4) years	Dep. & Anx. symptoms	Yes; females only	Self-reports every 3 months for 1-3 yrs while undergoing detoxification program	Mood symptoms, craving	Positive correlation between craving and 5 aspects of negative mood disturbance (fatigue, bewilderment, anxiety, depression, and hostility)
**Simpson et al**. (35)	124	None	N/R	N/R	Current psychiatric disorder in 53.2% females and 27.4% males	Yes	One-time self-report assessment	Psychiatric symptoms, perceived stress, coping strategies,	Female > male, childhood emotional and sexual trauma, psychiatric and drug problems, poorer treatment outcomes, current psychiatric disorder
***Brain-Based Findings***									
**Berman et al**. (36)	10	12	T1: 6.7(1.6) days T2: 27.6(.96) days	8.89(4.2) years	Dep. symptoms	No	PET, glucose metabolism	Dep. symptoms	MA>CTL: change in global GM; Within MA: ↑ GM in parietal regions, dep. symptoms neg. corr. w/ parietal GM
**Bischoff-Grethe et al**. (37)	17	23	173(160) days	N/R	No other substance abuse/dependence besides meth, nicotine, cannabis, alcohol	No	Cross-sectional, fMRI	Monetary loss	Loss anticipation – MA<CTL: VS, posterior caudate; MA only: loss>gains in anterior & posterior caudate
**Dean et al**. (38)	15	None	7.5(2.6) days	7.80(4.89) years	No Axis I diagnoses other than MA and nicotine dependence	No	RSFMRI within MA only	Dep. & anx. symptoms, ER	Within MA: amygdala-hippocampus RSFC pos. corr. w/ childhood maltreatment, dep., anx., ER & neg. correlated with self-compassion, mindfulness
**Kim et al**. (39)	19	19	20.5(8.3) days	13.6(7.3) years	None	No; males only	Cross-sectional, fMRI	Empathy task	MA<CTL: OFC, hippocampus, mean % correct answers on empathy task; MA>CTL: DLPFC
**Kim et al**. (40)	19	19	20.5(8.3) days	13.6(7.3) years	None	No; males only	Cross-sectional, fMRI	Emotion-matching task	MA<CTL: DLPFC, Insula; MA>CTL: fusiform gyrus, hippocampus, parahippocampal gyrus, posterior cingulate cortex
**London et al**. (41)	17	18	4-7 days	10.1(1.3) years	Dep. symptoms	No	PET, glucose metabolism	Dep. & anx. symptoms	MA>CTL: Dep. & anx. symptoms, GM in OFC, posterior cingulate, amygdala, ventral striatum, cerebellum; MA<CTL: GM in ACC, insula; Within MA only: Dep. symptoms pos. corr. w/ GM in amygdala & anterior cingulate gyrus, State/trait anx. neg. corr. w/GM in ACC & Insula
**Okita et al**. (42)	94 (27 PET)	102 (20 PET)	Among PET: 4.0(2.59) days	N/R	N/R	No	PET, dopamine	Emotion Regulation	MA>CTL: DERS total score; Across groups: DERS total score pos. corr. w/ amygdala D2-type receptor availability; MA only: DERS + corr. w/addiction severity
**Okita et al**. (43)	23	17	≥7.2(3.11) days	10.4(7.33) years	N/R	No	PET, dopamine	Alexithymia	MA>CTL: alexithymia; Within CTL: alexithymia; pos. corr. w/ D2-type receptor availability in ACC, Insula
**Payer et al**. (44)	25	23	9.91(4.57) days	11.4(7.8)	N/R	No	Cross-sectional, fMRI	Affect processing	MA<CTL: IFG during affect matching, Affect labeling – no group diff.
**Payer et al**. (45)	12	12	8.6(3.5) days	N/R	N/R	No	Cross-sectional, fMRI	Affect matching task	MA<CTL: VLPFC, fusiform gyrus; MA>CTL: dACC; Contrast: emotion match>shape match
**Payer et al**. (46)	53	47	N/R	11.0(7.7)	None	No	Cross-sectional, fMRI	Emotional faces viewing task	MA<CTL: VLPFC, DLPFC; MA>CTL: self-reported aggression
**Sekine et al**. (47)	11	9	7 days-1.5 years	1 month-15 years	Anxiety, depression, hallucinations	No, males only	PET, dopamine	Psychiatric symptoms	MA<CTL: DTD in nACC, PFC, caudate; MA only: severity of psych. symptoms pos. corr. w/duration of use, ↓DTD in caudate/nACC, neg. corr. w/ duration of MA use
**Sekine et al**. (48)	11	9	7 days-1.5 years	1 month-15 years	Anxiety, depression, hallucinations	No, males only	PET, dopamine	Psychiatric symptoms	MA<CTL: DTD in OFC, DLPFC, amygdala; Within MA: DTD in OFC, DLPFC neg. corr. w/ duration meth use & severity of psych symptoms
**Stewart et al**. (49)	20	22	45.47(19.76)	N/R	Comorbid alcohol (n=8), cocaine (n=2), cannabis (n=2), opiate (n=2) use disorders	No	Cross-sectional, fMRI	Loss and aversive interoceptive stimuli	MA>CTL: trait anxiety; MA<CTL; AI, IFG across trials, PI, ACC during aversive stimuli, ACC to punishment/loss & aversive stimuli
**Stewart et al**. (50)	18 relapsed MA	42 abstinent MA	33.9 ± 20.1 days	Relapsed: 13.3(8.9): Abstinent: 13.7(10.0)	Comorbid alcohol, cocaine, marijuana, nicotine use	No	Cross-sectional fMRI & longitudinal SU data	Loss	Relapsed<Abstinent – across win, loss, tie: insula, striatum, thalamus, posterior cingulate, precuneus; across loss and tie: AI
**Uhlmann et al**. (51)	21	19 MA-associated psychosis, 19 CTL	Median = 21 days, range 1-240 days	5.6(2.3) years	No other lifetime or current dx of psychiatric disorder	Yes; within MA insula cortical thickness M>F	Cross-sectional, structural MRI	ER self-report	MA>CTL: entorhinal cortex, insula cortical thickness; MA<CTL: overall ER skills
**Wei et al**. (52)	21	22	9.71(8.19) months	27.14(13.79) months	N/R	No, females only	ERP	Monetary loss	MA>CTL: FRN for loss vs. gain
**Yin et al**. (53)	26	26	≥ 24 h	Median=2.8 yrs	N/R	No	Cross-sectional, fMRI	Emotional faces vs. MA cue viewing task	MA cue images – MA>CTL: ACC; Emotional faces – MA<CTL: frontal lobe

ACC, anterior cingulate cortex; AI, anterior insula; CTL, control; dACC, dorsal anterior cingulate cortex; DERS, Difficulties in Emotion Regulation Scale; DLPFC, dorsolateral prefrontal cortex; DTD, dopamine transporter density; ER, emotion regulation; FRN, feedback-related negativity; F, female; fMRI, functional magnetic resonance imaging; f/u, follow-up; GM, glucose metabolism; IFG, inferior frontal gyrus; M, male; MA, methamphetamine; MDD, major depressive disorder; nACC, nucleus accumbens; N/R, not reported; OFC, orbitofrontal cortex; PET, positron emission tomography; PFC, prefrontal cortex; PI, posterior insula; RSFC, resting state functional connectivity; SU, substance; T1, time 1; T2, time 2; VLPFC, ventral lateral prefrontal cortex; VS, ventral striatum.

**Table 3:** Treatment studies involving negative reinforcement processes in methamphetamine users.

Author *(first author, year)*	Meth Group *(N)*	Comparison Group *(N)*	Meth Chronicity [*M*(*SD*)]	Comorbid Diagnoses	Gender Examined?	Study Design	Intervention	NR Variables	Results
***Psychotherapy Interventions***									
**Carrico et al**. (54)	55 HIV+ MSM randomly assigned to positive affect intervention	55 HIV+ MSM randomly assigned to attention-control	N/R	N/R	N/A	Pre- and post-intervention, 3-month f/u	Positive affect intervention vs. attention control delivered during CM	Negative and positive affect	PA intervention ↑ positive affect, mindfulness, ↓ craving, stimulant use
**Glasner-Edwards et al**. (55)	526	None	N/R	Depression	No	Longitudinal, 3 yr f/u	16-week Matrix Model: CBT, family edu. groups, support groups, individual sessions	Depression symptoms	Dep. severity ↓ treatment adherence Dep. at f/u **↑**MA use outcomes MA abstinence ↓ depressive symptoms Dep. ↑ overall impairment
**Glasner-Edwards et al**. (4)	526	None	N/R	Anxiety	No	Longitudinal, 3 yr f/u	16-week Matrix Model: CBT, family edu. groups, support groups, individual sessions	Anxiety symptoms	Anx. ↓ treatment adherence, ↑ family, medical, drug, psychiatric problems
**Glasner-Edwards et al**. (56)	526	None	N/R	34% with current dx of mood, anxiety, or antisocial personality disorders	Yes	Longitudinal, 3 yr f/u	16-week Matrix Model: CBT, family edu groups, support groups, individual sessions	Depression & anxiety symptoms	Anx. ↓ substance use outcomes, ↑ utilization of health services, ↑ psychiatric symptoms 3-years post-treatment
**Glasner-Edwards et al**. (57)	9 stimulant users assigned to MBRP intervention	13 stimulant users assigned to health education	N/R	N/R	No	Longitudinal, baseline and treatment end	8-week MBRP	Salivary cortisol stress response, subjective stress, anxiety, craving	MBRP ↓ salivary cortisol, subjective stress, anxiety, & craving in response to post-tx stress-test
**Hopwood et al**. (58)	94 tx completers	21 d/c tx	N/R	21% Dep., 17% phobias, 16% PTSD, 20% Borderline PD, 28% ASPD	No	Longitudinal, 30-180 days	Group therapy focused on functional analysis and relapse prevention + NA/AA techniques	Emotion regulation, negative emotionality	↑ ability to regulate negative emotions ↑ tx persistence -↓ negative emotionality ↑ tx outcomes
**Kay-Lambkin et al**. (59)	135 MA + comorbid depression	52 MA without depression	N/R	N/A	No	Baseline, 5 weeks, 6 months	Self-help book vs. 2 sessions CBT/MI vs. 4 sessions CBT/MI	Depression symptoms	DEP+>DEP: severity of MA use, change in MA use from baseline to 5 weeks DEP+ only: ↓ dep. at 5 weeks w/ 3-4 sessions
**Peck et al**. (60)	162 gay and bisexual men	None	8.34(5.9) years	73.2% mild or higher severity depression	N/A	16-week randomized clinical trial, 26- and 52-week f/u	Random assignment to: CBT, CM, CBT+CM, Gay-specific CBT	Depression symptoms	All participants reported ↓ MA use and dep. symptoms up to 1-yr post-tx, MA use in past 5 days predicted Dep. symptoms, Dep. symptoms did not predict MA use
**Polcin et al**. (61)	111	106	N/R	N/R	No	Baseline, 2-, 4-, 6-month follow-up	9-session Intensive MI vs. 1-session standard MI + 8 nutrition edu. sessions	Psychiatric problems and problem severity	Intensive MI only: psych. prob. ↓ days, ↓ psych. prob. Severity from baseline to 2-month
**Polcin et al**. (62)	111	106	N/R	N/R	No	Baseline, 2-, 4-, 6-month follow-up	9-session Intensive MI vs. 1-session standard MI + 8 nutrition edu. sessions	Depression symptoms	Across interventions: ↓ psych. prob. severity from BL to 2-month predicted ↓ use prob. severity
***Exercise Interventions***									
**Rawson et al**. (63)	69	66	N/R	N/R	No	Pre- and post-intervention	8-week structured exercise program vs. health education sessions	Depression & anxiety symptoms	Exercise intervention: ↓ Dep. & Anx. symptoms overall; Dose effect: ↑exercise sessions ↓ Dep. & Anx. symptoms
**Wang et al**. (64)	24	N/A	83.92(56.04) months	N/R	No	counterbalanced	Acute exercise session vs. active reading session	Craving	Acute exercise session ↓ craving
**Wang et al**. (65)	25	25	Exer.: 83.32(53.71) months Att. CTL: 83.92 (58.32)	N/R	No	Baseline, 6-week, post-tx	12-week RCT of aerobic exercise vs. attentional control	Craving	Exercise intervention ↓ craving
***Pharmacotherapy Interventions***									
**Cruickshank et al**. (66)	13	18	N/R	Elevated Dep. & Anx. Symptoms but specifics N/R	No	2 week randomized placebo-controlled, double-blind, trial of mirtazapine	Narrative therapy counseling + mirtazapine or placebo	Depression & anxiety symptoms, stress	No sig. group diff. for any symptom measure
**Elkashef et al**. (67)	79	72	Bupropion: 10.42(7.59) yrs Placebo: 9.97(6.10)	Dep. symptoms on HAM-D Bupropion: 19% Placebo: 21%	Yes	Randomized, placebo-controlled, double-blind trial of bupropion	Bupropion + group CBT vs. placebo + group CBT	Depression symptoms	No group differences in dep. symptoms or craving
**Heinzerling et al**. (68)	Baclofen: 25, Gabapentin: 26	Placebo: 37	Baclofen: 8.8(7.43) yrs Gabapentin: 10.12(6.28) yrs Placebo: 9.59(5.92) yrs	Dep. symptoms on BDI	No	16-week, randomized, placebo-controlled, double-blind trial of two GABAergic medications: baclofen & gabapentin	Relapse prevention groups + baclofen, gabapentin, or placebo	Depression symptoms	No sig. group diff. in craving, retention, or depression
**Shoptaw et al**. (69)	Sertraline + CM: 61, Sertraline only: 59	Placebo + CM: 54, Placebo only: 55	Sertraline + CM: 10.1(6.0) yrs sertraline only: 9.9(6.1) yrs placebo + CM: 8.7(5.4) yrs placebo only: 8.5(4.8) yrs	Depression symptoms on BDI	No	Randomized, placebo-controlled, double-blind trial	12-weeks: sertraline+CM vs. sertraline only vs. placebo+CM vs. placebo only	Depression symptoms, craving	No sig. effects of sertraline; sertraline contraindicated for MA dependence; CM: higher proportion of 3-weeks abstinence
**Shoptaw et al**. (70)	36	37	Buproprion: 11(9.6) yrs Placebo: 8.3(5.8) yrs	Depression symptoms on BDI	No	12-weeks longitudinal	Buproprion vs. placebo, in addition to CM+CBT	Depression symptoms, craving	No sig. diff. between bupropion and placebo on reducing dep. symptoms or craving
***Brain Stimulation Interventions***									
**Liang et al**. (71)	24 rTMS	24 sham rTMS	Real: 6.5(4.4) Sham: 8.5(4.2) days	Real: 4.6(3.0) Sham: 5.6(3.3) yrs	No, males only	10-sessions randomized, double-blind, controlled trial	10 Hz rTMS to left DLPFC	Depression & anxiety symptoms	Real rTMS: ↓ Dep. & Anx. symptoms, craving; Both groups: ↓ withdrawal symptoms → ↓ craving and ↓ anx. but not dep.
**Lin et al**. (72)	40 rTMS	40 sham rTMS, 25 waitlist/no treatment	Real: 197.10(16.87); Sham: 189.23(14.31); Wait-list: 190.08(15.17) days	Real: 8.30(.81); Sham: 7.15(.73); Wait-list: 8.92(1.22) yrs	No, males only	5 sessions/week for 6-weeks, randomized, double-blind, controlled trial	10 Hz rTMS to left DLPFC	Depression & anxiety symptoms	Real rTMS: ↓ dep. & anx. symptoms; Sham & CTL: ↑ dep. symptoms
**Su et al**. (73)	15 rTMS	15 sham rTMS	Real: 3.00(1.56); Sham: 2.80(1.70) months	Real: 40.33(32.04); Sham: 60.80(41.40) months	No, males only	5-session, randomized, double-blind, controlled trial	10 Hz rTMS to left DLPFC	Depression & anxiety symptoms	Real TMS: ↓ craving; Both groups: ↓ dep. symptoms, no change in anx. symptoms

ASPD, antisocial personality disorder; BDI, Beck depression inventory; CBT, cognitive behavior therapy; CM, contingency management; DLPFC, dorsolateral prefrontal cortex; f/u, follow-up; HIV, human immunodeficiency virus; MA, methamphetamine; MBRP, mindfulness-based relapse prevention; MI, Motivational interviewing; MSM, men who have sex with men; N/R, Not reported; PA, positive affect; PD, personality disorder; PTSD, posttraumatic stress disorder; rTMS, repetitive transcranial magnetic stimulation.

## Subjective, Behavioral, and Physiological Evidence for Negative Reinforcement

Depression and anxiety are two common negative affective states that have been found to have strong associations with MUD. Major Depressive Disorder (MDD) is characterized by depressed mood, anhedonia, sleep, and appetite disturbance, suicidal ideation/intent, extreme guilt, and difficulties in concentration and attention (22). Anxiety disorders are characterized by exaggerated worry and/or panic symptoms that are linked to distress and impairments in social, occupational, or other functioning (22). Initial or maintained use of methamphetamine may be motivated in part by the alleviation of symptoms related to depression or anxiety.

It is unclear whether symptoms associated with negative emotional states characteristic of MDD and anxiety disorders exist prior to methamphetamine use or develop only as a consequence of use. Pre-existing negative emotional states may initially motivate substance use only to be exacerbated by further use, or these emotional states may develop as a symptom of persistent methamphetamine consumption, tolerance, and withdrawal. It has been reported that 39% of methamphetamine patients have a history of anxiety disorders prior to methamphetamine initiation, while 76% of patients report anxiety symptoms after initiating use (2). A dose-dependent response has also been observed, with each additional day of methamphetamine use in the past 6 months corresponding to an increase in anxiety over that time window (74). MDD is also highly prevalent in methamphetamine users; for instance, approximately 40% of a sample of 400 current MUD entering treatment met diagnostic criteria for MDD. An additional 44% met symptom criteria for MDD, although the symptoms users were experiencing were better explained by consequences of psychoactive substance use (75). These findings clearly demonstrate the high prevalence of anxiety and depressive symptoms evident in MUD and demonstrate that these symptoms are often present prior to substance use initiation but can also be a consequence of use. These results are particularly concerning in light of research suggesting that among MUD, ineffective emotion regulation and coping strategies result in negative emotions and stress, which in turn are associated with drug use disorders, increased likelihood of relapse, and extended length of relapse periods (33).

Negative emotional symptoms are also a well-documented manifestation of methamphetamine withdrawal; after 1-7 days of abstinence, 34% of 210 MUD individuals report some symptoms of anxiety disorders ranging from mild to moderate (73). But with continued abstinence from methamphetamine (ranging from 6 days to 1 year), self-reported emotional symptoms of depression and anxiety appear to decrease among a cross-sectional sample of MUD (76). However, in a cohort of incarcerated women, lifetime MUD predicted current and past psychological symptoms, but lifetime psychological diagnoses did not predict lifetime drug use disorder or increased risk of use prior to incarceration (77). Taken together, these findings suggest that while depression and anxiety may predate MUD or remain persistent during abstinence for some individuals, for others these symptoms may be brought about or exacerbated by methamphetamine use and MUD. However, these studies rely solely on cross-sectional samples, and longitudinal studies are needed to determine the exact temporal relation between psychological symptoms and methamphetamine use.

### Subjective Evidence

The presence of negative emotional states such as depression and anxiety among MUD is hypothesized to be the manifestation of emotional dysregulation (26). It is thought that, in the absence of effective emotion regulation strategies, individuals with MUD may resume methamphetamine use to cope with life events, stress, or withdrawal and relieve negative affect (78–81). Based on self-report, MUD endorsed lower self-regulation and affective control compared to healthy comparison subjects (CTL) as well as individuals with problematic narcotic use (NA), although detailed characteristics were not provided about the substance using groups (25). Specifically, on a questionnaire developed to measure one’s ability to conceptualize and flexibly implement goal-directed behaviors, MUD reported lower levels on the subscales of receiving, triggering, searching, and formulating (4 out of 7 subscales) than both NA and CTL. MUD also reported lower affective control over angry, depressed, anxious, and positive emotions compared to NA and CTL (25). These findings suggest that affective regulation deficits may be unique to methamphetamine or stimulant users.

The role of negative reinforcement in perpetuating methamphetamine use was also explored by Newton and colleagues (24). Seventy-three non-treatment seeking MUD were surveyed to examine their reasons for continued substance use, which were categorized as positive reinforcement, negative reinforcement, or inhibitory control dysfunction (i.e., impulsivity). While questions pertaining to positive reinforcement or “pleasure seeking” (i.e., to experience a high) as an important motivator for continued use were endorsed more frequently, a significant proportion of the sample endorsed questions pertaining to negative reinforcement or “pain avoidance” (i.e., to reduce bad feelings or withdrawal symptoms; (24). Importantly, the majority of the sample endorsing negative reinforcement items perpetuating their drug use did not endorse questions related to positive reinforcement. This suggests that while positive reinforcement is commonly thought to play a larger role in maintaining substance use than negative reinforcement, there may be a unique subsample of substance users whose drug consumption is predominantly maintained by negative reinforcement processes.

### Behavioral and Physiological Evidence

Using behavioral measures, Chen and colleagues (26) examined the emotional response of 60 MUD, currently receiving treatment (abstinent 4.85 ± 1.12 months), and 30 CTL while viewing videos selected to elicit fear, anger, amusement, and joy. Self-reported emotional ratings were collected in conjunction with objective physiological measures of startle response and skin conductance. Skin conductance levels have been shown to reflect the arousal level of a stimulus, with an increase reflecting stress and excitement and a decrease reflecting relaxation (82). Startle response provides a measure of emotional valence, whereby negative emotional experiences exacerbate the startle response and positive emotional experiences reduce it (83). Overall, MUD compared to CTL reported lower levels of subjective arousal in response to fear videos but demonstrated higher levels of physiological arousal (startle response and skin conductance) to anger videos when compared to neutral videos (26). MUD also showed a greater level of skin conductance and lower level of startle response than CTL while viewing joy versus neutral videos. The higher objective response to anger videos demonstrated by MUD may be reflective of an increased negative emotional state and overall increased stress-response. The self-reported lower arousal levels in response to fearful stimuli among MUD may reflect an inability to accurately recognize and regulate withdrawal-related negative emotions, resulting in real-life difficulties in avoiding such stimuli and the continuation of drug-seeking behavior. MUD also differed from CTL in their physiological response to joyful videos. The increased level of skin conductance to joyful videos suggest that MUD find joyful stimuli more arousing than CTL, while the dampened startle response to joyful stimuli in MUD compared to CTL suggests that their evaluation of positive emotional stimuli is blunted. This finding is contrary to expectations, given that MUD is conceptualized as involving a blunted response to non-drug related positive stimuli. However, this may reflect a cognitive bias towards negative stimuli within MUD wherein positive stimuli evoked a greater response from MUD than CTL on a measure of arousal, but the reduced startle response among MUD compared to CTL may suggest an inability to assess the positive value of natural rewards. Again, the cross-sectional nature of this study limits the conclusions that can be drawn about the temporal relationship between substance use and emotion dysregulation; however, the results clearly demonstrate altered emotional processing in MUD relative to CTL.

Deficits in emotional processing are also thought to relate to impaired social cognition among MUD. A facial affect recognition task has been used to demonstrate this deficit in MUD abstinent for an average of 6 months (27) as well as MA/MUD abstinent an average of 20 days (28). In both of these studies, individuals who used methamphetamine demonstrated a decreased ability to correctly match faces based on the expressed emotion. Similar results were found among MUD in relation to social emotional cognition and problem solving; at enrollment, MUD (abstinent from MA < 3 months) performed worse than CTL on a task requiring individuals to identify different facial expressions, as well as on a maze learning task assessing problem-solving skills (29). However, this same study reported that, at re-test 6 months later, MUD demonstrated improved social emotional cognition and problem solving compared to CTL using the same tasks. These results suggest that methamphetamine users may experience difficulties and be uncomfortable in social interactions because they cannot accurately read and respond to a speaker’s emotional state (27, 28) and lack the skills needed to resolve these issues (29). These social deficits may be a risk factor for additional use, as methamphetamine can acutely lessen social anxiety and irritability (28, 32). However, continued abuse may cause interpersonal problems due to misunderstandings, resulting in stress and negative mood states (84). This then leads individuals to use methamphetamine again to alleviate this discomfort, ultimately resulting in a negatively reinforcing cycle of use and uncomfortable sensations.

### Sex-Specific Findings

A number of studies have specifically focused on examining methamphetamine use among females, providing evidence for gender-based differences. Such studies have shown strong relationships between negative emotions and drug craving among female users, as well as deficits in coping. Among 113 female methamphetamine users participating in a compulsory detoxification program (average detoxification duration of 8.7 ± 4.8 months), craving level positively correlated with negative mood disturbances including fatigue, bewilderment, anxiety, depression, and hostility after controlling for each user’s weekly dose of methamphetamine (34). Alternatively, among a sample of 203 non-treatment seeking methamphetamine users, the opposite pattern was observed, wherein depression and anxiety symptoms positively correlated with methamphetamine craving among men, but not women (31). This difference in findings may be related to use status at the time data were collected, given that two studies reported on abstinent users (34, 73) and one examined current users (31).

Gender differences have also been observed among self-reported reasons for use. Maxwell and colleagues (32) conducted a large survey with 222 methamphetamine users to better understand motivations for drug consumption. According to this survey, in addition to accomplishing tasks and losing weight, women also reported using methamphetamine to feel less depressed, suggesting they may have difficulty regulating their emotions in other ways even prior to initiation of methamphetamine use. This potential deficit in emotion regulation was examined behaviorally among 30 females with MUD (abstinent an average of 8.68 ± 3.64 months) and 30 CTL females using musical stimuli (30). In comparison to CTL, female MUD reported lower arousal ratings and showed an inhibited startle response to fearful music. Female MUD also reported lower arousal than CTL in response to happy music. These findings demonstrate that, within a sample of female patients, MUD have an altered perception of emotional stimuli regardless of valence relative to CTL. Additionally, female methamphetamine users endorse higher emotional and sexual childhood trauma than male users (35), and it has been hypothesized that women may use substances as a method of coping with these past traumas. In line with this hypothesis, women with MUD reported higher-levels of emotion-focused coping, including substance use, than both women CTL (33) and men with MUD (35), while men and women with MUD report comparable low levels of problem-focused coping (35). However, neither of these studies reported important use characteristics (i.e., chronicity of use, duration of abstinence) that may influence one’s ability to cope. Despite this limitation, these findings strengthen the hypothesis that MUD may administer methamphetamine as a form of self-medication to relieve uncomfortable mood and body-relevant sensations, thereby negatively reinforcing methamphetamine use; this relationship may be stronger in female than male patients. Therefore, female methamphetamine users may be more prone than male users to turn to substance use to cope with uncomfortable emotional distress.

### Conclusions

The literature demonstrates that emotional processing is dysfunctional among MUD and supports the hypothesis that methamphetamine use is not only reinforced by its rewarding euphoric effects but also by its relief of negative and uncomfortable effects. Specifically, methamphetamine use appears to relieve symptoms of anxiety and depression that may or may not be pre-existing. These psychiatric symptoms are often worsened when individuals try to reduce or abstain from use, leading individuals to crave methamphetamine to alleviate these uncomfortable feelings. MUD is also associated with deficits in emotional processing. These deficits relate to the processing of positive and negative stimuli and methamphetamine use may help to reduce the exaggerated response to negative stimuli and alter the lack of response to natural rewards. Overall, the data support the conclusion that negative reinforcement, not just positive reinforcement, is an important factor in the perpetuation of substance use and suggests that learning to use healthy coping skills to address these symptoms in lieu of substance use may improve treatment outcomes. However, the extent to which negative reinforcement contributes MUD over positive reinforcement remains unclear.

## Brain-Based Evidence for Negative Reinforcement Mechanisms

The three-stage model of addiction coincides with dysfunction in brain systems implicated in reward, stress, and executive function (9). The initial stage of binge/intoxication is driven by the acute reinforcing effects of stimulant use, which activate and alter dopamine transmission in brain regions associated with reward including the ventral tegmental area and nucleus accumbens (7). With prolonged use, these changes in neurocircuitry are thought to interact and alter other brain networks implicated in executive functioning (frontal regions), emotion regulation and stress responsivity (amygdala and hypothalamus), and interoception (insula and ACC). Prolonged use also results in the attribution of incentive salience to previously neutral cues that have become paired with drug use, and a conditioned response to continue seeking drugs of abuse. This neural change involves striatal regions and ultimately effects synaptic changes in glutamate transmission within PFC and amygdala (9). This in turn results in reduced executive functioning and increased drug-seeking behavior.

The binge/intoxication stage is followed by a stage of withdrawal/negative affect characterized by irritability, emotional discomfort, stress, and alexithymia (9). With prolonged exposure, the rewarding effects of the drug decrease as reflected by hypoactivation within reward regions (e.g., ventral striatum) and over-active stress-systems reflected by amygdala hyperactivation (8). This evolves into the third stage of preoccupation/anticipation, a key contributor to relapse. Altered functioning within frontal regions results in executive dysfunction when presented with a salient cue signaling substance use. Such cue-induced craving has been observed to activate regions including dorsolateral PFC (DLPFC), ACC, OFC, and hippocampus. These deficits in executive function impact decision making, self-regulation, and inhibitory control, resulting in an inability to inhibit maladaptive behaviors and continued drug-seeking behaviors despite negative consequences.

Overall, the three-stage model of addiction describes a cycle wherein initial positive reinforcement of substance use evolves to include negative reinforcement as the rewarding effects of the drug decrease and uncomfortable emotional and stress responses emerge. The decreases in prefrontal executive function may exacerbate these effects by reducing one’s ability to control responses to negative reinforcement mechanisms. This cycle is reflected by alterations in brain functioning within regions involved in reward (striatum), regulation of emotions and stress (amygdala and hypothalamus), interoception (insula and ACC), and executive functioning [various frontocingulate regions; Koob (7), Koob and Volkow (9), Volkow et al. (8)] . By examining the existing brain-based literature on negative reinforcement, the goal is to determine the state of the evidence supporting the three-stage model of addiction and to highlight regions that can possibly be targeted by intervention to improve substance use outcomes. Details of the studies outlined below can be found in **Table 2**.

### Task-based fMRI

Given the relationship between substance use and emotional processing deficits, fMRI studies focused on the experience and processing of emotion in MUD allow for the examination of negative reinforcement mechanisms. Such paradigms include facial affect tasks, which require individuals to match, label, or view emotional stimuli. Other tasks involve performance errors, loss of reward, or perturbations in interoception, defined as the sensing and processing of information signaling the internal state of the body (85). These types of tasks elicit negative and uncomfortable states and sensations and allow for the comparison of CTL and MUD during these experiences in order to draw conclusions about negative reinforcement. Additionally, these types of paradigms have been demonstrated to activate brain regions thought to be implicated in the three-stage model of addiction (7, 9).

Emotion processing among abstinent MUD has been evaluated using an empathy task (39) and emotion matching tasks (40, 43, 44). In response to viewing scenarios designed to evoke an empathetic response, CTL showed greater activation than MUD (abstinent 20.5 ± 8.3 days) in OFC and hippocampus (39), in line with previous findings of abnormal brain functioning among methamphetamine users within OFC, a region associated with social cognition (86, 87). However, MUD showed greater activation than CTL in DLPFC to these empathic scenarios (39), a region previously shown to be underactive in MUD during a two-choice response task involving varying levels of error feedback (87). Coupled with the lower mean percentage of correct answers on the empathy task among MA compared to CTL, this increased DLPFC activity in MA may be reflective of greater cognitive effort in light of inefficient processing of empathy.

Contradictory findings have been found using paradigms requiring individuals to match facial expressions varying on positive and negative valence. While performing an emotion matching task utilizing fearful and threatening images, MUD (abstinent 20.5 ± 8.3 days) demonstrated reduced activation in DLPFC and insula, as well as increased activation in fusiform gyrus (facial processing) and hippocampus relative to CTL (40). Alternatively, using a similar emotion matching task, MUD (abstinent 8.6 ± 3.5 days) showed reduced activation in the inferior frontal gyrus [IFG; Payer et al. (44)] and ventrolateral prefrontal cortex (VLPFC), regions implicated in affect processing, as well as fusiform gyrus (45). Compared to CTL, MUD also demonstrated greater activation in dorsal ACC, a region implicated in social distress, which was associated with increased hostility and interpersonal sensitivity amongst MUD (45). This finding may suggest that individuals with MUD are more susceptible to socially threatening cues. An attenuated response in VLPFC/DLPFC and other frontal regions in MUD compared to CTL has also been observed as a result of simply viewing emotional images [Payer et al., (46), Yin et al., (53)]; however, one of these studies did not report duration of abstinence (46), while the other only required a minimum of 24 h abstinent for inclusion and did not report specific abstinence/illness duration details [Yin et al. (53)], weakening the strength of the conclusions that can be drawn from these results.

These studies all demonstrate altered functioning in various frontal regions (DLPFC, VLPFC, OFC) and hippocampus in MUD compared to CTL, however, the activity patterns are in varying directions. These contradictory findings may be related to the type of emotional task used, as one requires individuals to identify empathetic responses while the other may elicit fear. In relation to negative reinforcement, these findings do suggest that MUD have disrupted processing of socio-emotional information, a pattern which could potentially contribute to their experience of negative mood states and inability to engage in adaptive behaviors. Future research that ties brain activation patterns to real-life function (i.e., neuropsychological functioning, theory of mind tasks, or other performance measures) would be helpful to provide insight into the exact functional role of various brain regions.

Tasks involving loss can also be used to examine negative reinforcement processes among methamphetamine users. Differential response to loss in MUD compared to CTL could suggest that methamphetamine users experience aversive outcomes differently, which could contribute to relapse. For instance, a stronger (more exaggerated) brain and/or behavioral response to loss among methamphetamine users may negatively reinforce the decision to continue to use stimulants in order to relieve uncomfortable sensations associated with this loss. Bischoff-Grethe and colleagues (37) demonstrated this relationship using a probabilistic feedback expectancy task that allowed for the examination of anticipation and receipt of monetary gains and losses. MUD (abstinent 173 ± 160 days) showed lower ventral striatum signal than CTL when anticipating loss but greater signal in the caudate in response to the experience of loss compared to reward, while CTL did not show a differential response based on outcome (37). Ventral striatum is implicated in anticipating potential reward and loss (88, 89) and the caudate is involved in goal-oriented behavior as it receives projections from the frontal cortex (90). Together, this blunted response to the anticipation and experience of loss in MUD may contribute to the poor decision-making that is characteristic of this population, and continued drug-use despite negative consequences (37).

Loss has also been shown to elicit reduced activation in regions implicated in processing reward and interoceptive signals among a sample of relapsed MUD. Sixty MUD (abstinent 33.9 ± 20.1 days) enrolled in a treatment program completed a rock-paper-scissors task during a baseline fMRI session (50). One year later, MUD were categorized as abstinent (*n* = 42) or relapsed (*n* = 18). Examination of the baseline fMRI data revealed that those who relapsed over the follow-up period, compared to those who remained abstinent, had initially exhibited decreased activation in insula and striatum across winning, tying, and losing outcomes. Relapsed MUD also showed significantly lower anterior insula activation specifically to ties and losses than abstinent MUD. These findings suggest that altered activity in brain regions known to be dysfunctional in MUD may be able to be examined prospectively as a potential marker of poor treatment outcomes, such as relapse. These findings are somewhat contradictory to previously reported findings as the altered brain functioning was found across all outcomes (i.e., win, loss, tie). Regardless, these results suggest there may be underlying differences in the neural processing of situational outcomes that put an individual at greater risk for continued substance use problems.

This altered response to loss has also been demonstrated among MUD while simultaneously experiencing an aversive interoceptive manipulation. Interoceptive processing is the ability to sense the internal state of the body and engage in goal-directed behaviors to maintain equilibrium (91). Researchers have suggested that this interoceptive system is altered in addiction, resulting in a bodily prediction error, whereby a discrepancy between one’s predicted internal state and the actual internal state experienced may result in an increased propensity towards substance use in an attempt to regain balance of the internal state (49, 92). Among CTL, ACC is implicated in this process of registering and initiating motivated actions to restore balance, while cognitive control frontal regions, including IFG, contribute to decision-making processes. However, in MUD ACC and IFG have been shown to be underrecruited, likely resulting in an inaccurate representation and limited adaptive behaviors to address potential prediction errors. Using a two-choice prediction task with fixed error rates, Stewart and colleagues (49) demonstrated that the experience of loss paired with an aversive interoceptive manipulation (anticipation and experience of loaded breathing) elicited greater ACC response in CTL than MUD (abstinent 45.47 ± 19.76 days), suggesting MUD may be underrecruiting this brain region to help manage this uncomfortable experience and adjust behavior accordingly (49). In comparison to CTL, MUD also exhibited reduced anterior insula and IFG activity across all trials and reduced posterior insula and ACC during breathing load trials regardless of outcome. Anterior and posterior insula differ functionally; posterior insula receives input about the physiological state of the body from other brain regions, such as the thalamus, and then passes this information on to the anterior insula to be further integrated with additional information and motivate the initiation of goal-oriented behaviors to regain homeostasis. Together, these results suggest that MUD have an altered response to unpleasant outcomes and physical stimuli compared to CTL and that they may lack the executive functioning resources needed to engage in goal-directed behaviors to help regulate the effect of unpleasant outcomes. Therefore, drug use may be negatively reinforced because of its ability to alleviate discomfort associated with unpleasant outcomes in the face of limited resources which hinder one’s ability to engage in alternative healthy forms of coping.

Overall, the fMRI literature reveals altered neural function in brain regions associated with emotion-processing, loss of reward, and interoception, including frontal regions, insula, ACC, and striatum. Interestingly, there is a lack of fMRI findings linking amygdala impairments to MUD/MA. Given amygdala’s role in emotion processing, it would be expected to play a crucial role in negative reinforcement processes. However, no identified study reported functional deficits in this region despite the use of emotion-matching tasks. Future research using tasks that elicit stress or fear responses, may help elucidate amygdala’s role in perpetuating methamphetamine use. While the direction of current findings is somewhat mixed between studies, interventions that aim to modify activity in the identified regions, and the behaviors associated with those regions, may hold promise for improving substance use outcomes.

### Resting-State fMRI

Differences between MUD and CTL have also been found using resting state fMRI. While task-based fMRI examines changes in blood flow within specific brain regions while an individual completes a task, resting state functional connectivity (RSFC) examines the temporal dependence of neuronal activity patterns between brain regions while at rest (93). In other words, while undergoing a resting state scan, individuals are not performing a task but instead are typically asked to relax and not think of anything in particular. Analyses then indicate the amount of correlation between activation within various regions to yield a measure of functional connectivity, suggesting the degree of communication and information processing between these regions. Within MUD, RSFC has revealed altered functional patterns compared to CTL. For instance, RSFC between amygdala and hippocampus was found to positively correlate with self-reported depression, trait anxiety, and emotion dysregulation within 15 abstinent (7.5 ± 2.6 days) MUD enrolled in a pilot study (38). Amygdala-hippocampus RSFC was also positively associated with self-reported childhood maltreatment. Together, results may indicate that traumatic experiences in childhood contribute to differences in brain functioning that are in turn associated with negative emotional states and dysfunctional emotional processing in adulthood. Longitudinal research is needed to test the hypothesis that childhood maltreatment as well as other negative or traumatic childhood experiences may foster the development of MUD as a form of emotion-regulation. Negative reinforcement may play a critical role in the development and maintenance of MUD among individuals who may be experiencing negative emotionality prior to substance use initiation as well as those who experience it as a consequence of use.

### Structural MRI

Structural brain differences among MUD have also been examined in relation to emotion processing. Cortical thickness was examined in relation to affect regulation abilities among 21 MUD abstinent for 1-240 days (median = 21 days) and 19 CTL, as well as 19 patients with methamphetamine-associated psychosis (51). When comparing MUD and CTL only, MUD were found to have higher cerebral thickness than CTL within insula and entorhinal cortex, a region involved in translating exteroceptive information. Self-report data on emotion regulation capabilities were also gathered using the *Emotion Reactivity Scale* (ERS) and *Difficulties in Emotion Regulation Scale* (DERS). These data revealed that MUD, relative to CTL, reported significantly greater difficulties with emotion regulation based on the ERS total scale and all subscales (*d* = 0.77-0.87), as well as difficulties with understanding emotions (*d* = 0.70) and impulse control (*d* = 0.81) on the DERS scale. However, none of the self-reported differences in emotion regulation reported by MUD correlated with the observed differences in cortical thickness (51). These findings demonstrate the presence of emotional dysregulation in MUD but do not suggest a link with brain structural abnormalities, thereby limiting the conclusions that can be drawn regarding the role of greater insula cortical thickness in methamphetamine addiction.

### Conclusions for MRI Findings

Brain regions that are repeatedly represented in the literature on MUD include various frontal regions (VLPFC, DMPFC, IFG, OFC), insula, hippocampus, and ACC (see **Table 2**). Taken together, differing patterns of brain activation in these regions compared to CTL suggest an altered experience of negative outcomes and an inability to regulate or respond in effective ways. Specifically, fMRI data demonstrates that MUD experience negative outcomes more intensely as reflected by an exaggerated response compared to CTL in reward-relevant brain regions [caudate; Bischoff-Grethe et al. (37)] and that they are unable to activate regions (ACC) necessary for regulating their response to negative outcomes (49). MUD show deficits in various frontal regions which are implicated in the ability to recognize and comprehend emotionally salient information and to produce mental representations regarding the internal states of others, suggesting that MUD lack emotional insight (44). Deficits in frontal regions may also contribute to one’s ability to integrate socio-emotional information and in turn regulate behavioral responses by inhibiting behaviors that are no longer useful [Payer et al. (44), Yin et al. (53)]. Hippocampus and ACC also play a crucial role in one’s ability to incorporate information and regulate a response. ACC monitors conflict and is overactive in MUD compared to CTL while viewing images of substance [Yin et al. (53)] and socially threatening situations (45). Taken together, these findings suggest that MUD are hyper-sensitive to these types of cues, which may stem from an inability to respond appropriately given altered hippocampal functioning which aids in the ability to incorporate previous experience and update response patterns accordingly (39, 40).

General findings on structural differences in MUD have been mixed, reporting both higher and lower cortical thickness in MUD than CTL (51). In relation to negative reinforcement, structural differences within insula and entorhinal cortex did not correlate with any measure of emotional regulation. Alternatively, results from a pilot study utilizing RSFC show more promise for identifying potential treatment targets to decrease psychological difficulties. Connectivity between amygdala and hippocampus appears to correlate with depression, anxiety, and emotion dysregulation, symptoms commonly reported among MUD (38). This suggests that amygdala-hippocampus connectivity may contribute to emotional regulation, and interventions that aim to strengthen the connection between these regions may be effective at breaking the cycle between experiencing negative affect and using methamphetamine to alleviate those symptoms.

A few limitations must be considered when interpreting data from MRI studies. First, the studies reported here predominantly consist of sample sizes with less than 25 per group (MUD vs. CTL), with the exception of three over 30 (44, 46, 50). Additionally, these studies were cross-sectional in nature and do not allow for the examination of the temporal relationship between brain functioning and methamphetamine use. Similarly, there is wide variation between and within studies with regards to duration of methamphetamine use and abstinence (see **Table 2** for abstinence/chronicity details). Reported abstinence ranged from 24 h [Yin et al. (53)] to 330 days (37) across studies, with one study reporting a range of 1-240 days (51). Duration of use was also quite varied, ranging from 3.3 to 20.9 years (see **Table 2**). Duration of abstinence and regular use are important variables in the substance use literature as they can have profound effects on the deficits observed. Without some consistency in these variables, at least within study, it is difficult to draw strong conclusions about the role of negative reinforcement in MUD. Further, only one of the studies presented here examines whether the observed deficits predict relapse or other treatment-related outcomes (50). Lastly, studies eliciting an emotional response were conducted within an experimental setting, suggesting the MUD could possibly respond differently in real-life personal situations. Overall, fMRI studies support the conclusion that MUD have an altered experience of emotional stimuli relative to CTL based on self-report and behavioral data. This deficit may make it difficult for MUD to understand their own bodily sensations and emotional responses as well as those of others. This may result in increased negative mood and stress, and ultimately reinforce the decision to use methamphetamine given its attenuation of these symptoms.

### Event-Related Potentials

Analysis of EEG data in the time domain yields an event related potential (ERP), a time-locked, electrophysiological response of the brain to a stimulus (94, 95). There are various ERP components, each with a unique electrophysiological profile and originating brain region. The feedback-related negativity (FRN) component is thought to originate within ACC and is described as a negative deflection in response to feedback onset. The reinforcement learning-error related negativity theory posits that the FRN is a reflection of a discrepancy between current outcomes and the expected result; in other words, this component peaks when outcomes are contrary to expectations. Compared to CTL (*n* = 22), individuals with MUD (*n* = 21; abstinent 9.7 ± 8.19 months) demonstrated an enhanced FRN in response to monetary loss versus gain during a gambling task (52), suggesting MUD have a stronger response to unanticipated loss. This was the only ERP study identified that examined negative reinforcement principles among MUD, and the results are difficult to reconcile with the previously discussed fMRI findings. Stewart and colleagues (49), showed MUD compared to CTL to have a reduced ACC response to punishment paired with an aversive interoceptive stimulus, while Bischoff-Grethe and colleagues (37) reported an exaggerated response within caudate to loss versus reward. These findings all suggest that MUD respond to loss/punishment differently than CTL but our understanding of exactly how they differ remains unclear. In relation to negative reinforcement, one factor that may contribute to these altered brain response patterns is depressive symptoms. Depressive symptoms were not reported in relation to ERP results (52) but MUD reported significantly greater depressive symptoms than CTL in the fMRI studies (37, 49). Future research, utilizing ERP and fMRI, should examine whether depressive symptoms contribute to an exaggerated response to loss/punishment among MUD. Overall, this altered response to negative outcomes and inability to modify behaviors accordingly may contribute to relapse.

### Positron Emission Tomography

As described above, the initial binge/intoxication stage of addiction alters neurotransmission in brain regions implicated in executive functioning (frontal regions), emotion regulation and stress responsivity (amygdala and hypothalamus), and interoception (insula and ACC). Dopamine plays a central role in the development and maintenance of substance use disorders. Even with initial use, methamphetamine alters neurotransmission of dopamine in reward areas [i.e., nucleus accumbens; Koob (7)], and with sustained use, these alterations can extend to regions of executive functioning (i.e., PFC) and emotion regulation (i.e., amygdala). PET allows for the examination of neurotransmission and has been employed in conjunction with measurement of emotional and psychiatric functioning within MUD.

Using PET techniques, widespread dopaminergic dysfunction has been demonstrated during periods of substance use and abstinence. Specifically, during early abstinence (4 ± 2.59 days), greater difficulties with emotion regulation reported by MUD than CTL was found to positively correlate with D2-type dopamine receptor availability within amygdala (42). This finding is in line with previous evidence suggesting that D2-type receptors in amygdala are thought to contribute to enhanced neural activity associated with a negative emotional state (96). Emotional dysregulation also positively correlated with severity of drug use as measured by the *Addiction Severity Index*, highlighting the role of negative affect in MUD. MUD (abstinent ≥7.2 ± 3.11 days) also reported greater alexithymia than CTL, but this did not relate to dopamine transmission in MUD. Instead, self-reported alexithymia positively correlated with higher D2-type receptor availability in ACC and insula, regions implicated in emotion processing and awareness of internal states, within CTL only (43). Taken together, these findings may indicate that altered dopamine transmission is associated with MUD’s difficulties regulating emotions but does not contribute to other difficulties observed in MUD.

MUD also showed reduced dopamine transmission within brain regions implicated in reward (i.e., nACC, caudate) and cognitive control (i.e., PFC, OFC, DLPFC). Specifically, among MUD (abstinent 7 days to 1.5 years), dopamine transporter binding potential in caudate and nACC negatively correlated with duration of methamphetamine use and overall psychiatric difficulties as measured by the *Brief Psychiatric Rating Scale* (47). Further, reduced dopamine transporter density in OFC and DLPFC negatively correlated with duration of methamphetamine use as well as severity of psychiatric symptoms (48). Although the results regarding regions of reward and cognitive control appear consistent, they were found within the same, relatively small, sample of 11 MUD and 9 CTL (47, 48). The conclusions that can be drawn regarding these findings are severely limited by the characteristics of the sample. MUD ranged in duration of use from 1 month to 15 years and duration of abstinence from 7 days to 1.5 years (see **Table 2**). Given that cessation of methamphetamine use is known to result in acute withdrawal during the first 24 h and subacute withdrawal for the first two weeks, the participants in this study were in varying stages of recovery. Similarly, 2 of the 11 MUD patients reported using methamphetamine for 6 months or less; it is highly likely that these individuals differ in important ways from the individuals reporting 12-15 years of regular use.

PET also allows for the examination of glucose metabolism, which is necessary for the process of neurotransmission (97). Given the importance of the first week of abstinence, two studies have employed PET to examine glucose metabolism in relation to psychiatric symptoms among MUD during this crucial time period. Both of these studies found altered glucose metabolism in MUD within reward, executive function, and emotion-processing regions. Importantly, these changes were found to correlate with self-reported mood symptoms, wherein depressive symptoms positively correlated with glucose metabolism within amygdala and ACC (41) but negatively correlated with glucose metabolism within left parietal lobe, a region which has previously been shown to have functional abnormalities among MUD (36). Anxiety on the other hand, was found to negatively correlate with glucose metabolism within insula and ACC (41). These findings highlight the altered brain function present among MUD in regions of emotion regulation and how this dysfunction correlates with the actual experience of altered mood. Emotion regulation interventions may help prevent newly abstinent individuals from relapsing and thereby negatively reinforcing their use by alleviating the uncomfortable sensations associated with emotional dysregulation and heightened negative affect.

Overall, the findings from PET studies point towards the importance of targeting emotion regulation skills during early abstinence. In line with negative reinforcement principles, the first week of abstinence is an important determinant of treatment engagement, retention, and outcomes, as MUD patients typically experience physical discomfort, depression, anxiety, and craving, often resulting in relapse as an attempt to reduce these uncomfortable sensations (36). Alterations in dopaminergic transmission and glucose metabolism in MUD appear to contribute to the presence and severity of symptoms related to emotional dysfunction, substance use, and psychiatric distress. These studies lend further evidence to suggest that altering amygdala activity or enhancing emotion regulation strategies may improve MUD treatment outcomes.

## Implications for the Treatment of Methamphetamine Addiction

Various treatments for problematic methamphetamine use exist, with varying effects on treatment outcomes of interest, including reductions in amount or frequency of substance use, duration of abstinence post-treatment, and alleviation of psychological symptomology (98). Based on the evidence outlined above, the experience of negative or uncomfortable sensations and emotions often contributes to methamphetamine use and maintenance; leaving these symptoms untreated may place individuals at greater risk for relapse (98). Therefore, interventions aimed at alleviating these symptoms may improve treatment retention and outcomes and prevent relapse (see **Table 3** for further details of studies outlined below).

### Psychotherapy Interventions

The primary psychotherapy interventions that have been examined for MUD patients include cognitive behavioral therapy (CBT), contingency management (CM), motivational interviewing (MI), and mindfulness-based relapse prevention [MBRP; Lee and Rawson, (98)]. Treatment can be provided on an outpatient basis or through a more intensive inpatient program. Programs vary in terms of duration, number of sessions, and required activities. Regardless, overall treatment outcome is typically measured in terms of abstinence rather than improvement of psychological symptoms, emotion regulation, or coping strategies. However, the role of these psychological and emotional factors in reinforcing methamphetamine use suggests that interventions targeting these symptoms could help improve substance use outcomes.

CBT, CM, MI, and MBRP are evidence-based psychotherapies that have been examined for the treatment of substance use disorders. CM is a form of operant conditioning wherein reductions in use or abstinence are reinforced by the delivery of some type of incentive (99). This approach utilizes positive reinforcement and can effect change by teaching patients new patterns and replacing previously reinforced patterns of substance use with new healthy patterns of behavior (99). CBT on the other hand, focuses more on helping patients better cope and respond to uncomfortable thoughts or emotions they may experience (100). Similarly, this helps patients learn new, healthy ways of coping to replace their previously patterns of using substances to cope with uncomfortable thoughts, emotions, or sensations. MI focuses on increasing a patient’s readiness for change by eliciting their own motivation for enacting change and by exploring any ambivalence they may have (101). It is often conducted over 1-2 sessions in preparation for more intensive treatment but can also be used as a stand-alone treatment for substance use reduction. Lastly, MBRP focuses on stress reactivity and negative affect in relation to drug craving. By using mindfulness techniques, patients learn to focus on the present and cope with discomfort without the use of substances (57). All four of these interventions have been examined within substance using populations and the following studies demonstrate the importance of treating co-occurring psychological symptoms in conjunction with substance use treatment.

Based on the hypothesis that methamphetamine use is reinforced by the relief of negative emotional symptoms, it is logical that interventions should aim to alleviate these emotional symptoms to promote substance use reduction or abstinence. This is supported by the finding that depression severity predicted poorer treatment adherence in a study of 526 MUD patients undergoing psychosocial treatment [*β* = -0.18, *SE* = 0.07; *p* = 0.01; Glasner-Edwards et al. (55)] . Similarly, among the same cohort, anxiety disorders predicted poorer substance use outcomes, increased utilization of health services, and greater levels of psychiatric symptoms 3-years post-treatment (4, 56). Taken together, these data highlight the effect of negative emotional symptoms on substance use and emphasize the need for psychiatric intervention in substance treatment programs.

Polcin and colleagues (61, 62) examined the relationship between psychiatric symptoms and substance use problems among 217 MUD patients randomized to receive either an intensive nine-session MI intervention or a single session of standard MI paired with eight nutrition education classes. Overall, both interventions resulted in reduced methamphetamine use and severity of use-related problems. However, only patients in the intensive MI group reported fewer days with psychiatric problems (other than depression and anxiety) and reduced severity of these problems (61). With further examination, across both interventions, changes in psychiatric problem severity from baseline to 2-month follow-up were found to predict changes in the severity of methamphetamine use-related problems, but not in the number of days individuals used substances (62). This relationship persisted through 6-month follow-up. Depression specifically has also been examined in the context of treatment for methamphetamine use. Methamphetamine users (MA and MUD; *n* = 214) with and without depressive symptoms were randomized to receive either a self-help book, or two or four therapy sessions consisting of MI and CBT (59). At baseline, individuals reporting depressive symptoms endorsed more severe methamphetamine use and drug-related problems than those who were not depressed. These depressed individuals also had greater change in methamphetamine use and depressive symptoms at 5-week follow-up, but unfortunately improvements were not sustained through 6-month follow-up. Importantly, these results suggest that methamphetamine use and depression are highly intertwined, and that methamphetamine-focused treatment may not be sufficient for long-term reduction of depressive symptoms, putting these individuals at increased risk for relapse. Overall, these results highlight the complex negatively reinforcing relationship between psychiatric problems and methamphetamine use. Additionally, MI appears to be an effective treatment for psychiatric symptoms and methamphetamine use but these effects may not be long-lasting.

Emotion regulation capacity has also been shown to predict treatment adherence. One hundred fifteen MUD patients enrolled in a residential substance use treatment program were followed from treatment entry and classified on the basis of whether or not they discontinued treatment early (58). Measures related to emotion regulation were collected including the DERS [Gratz and Roemer, (102)] and *The Multidimensional Personality Questionnaire Negative Emotionality Scale* [MPQ-NEM; Tellegen and Waller, (103)] . Overall, greater emotion regulation capacity at the time of treatment entry was associated with persistence through treatment (MPQ-NEM: *d* = .70; DERS: moderate to strong effect, *d≤*.70). MUD who discontinued treatment early reported lower emotional clarity, decreased ability to engage in goal-directed behavior despite emotional distress, and higher trait negative emotionality than those who completed treatment (58). Contrary to previous research showing motivation to be a predictor of treatment persistence (104), level of motivation as assessed by *The Stages of Change Readiness and Treatment Eagerness Scale* [SOCRATES; Miller and Tonigan, (105)] was unrelated to whether or not MUD discontinued treatment. Therefore, emotional regulation capacity may be a more important contributing factor to treatment success than motivation for treatment alone. This demonstrates the importance of addressing emotion regulation skills to help substance use patients better cope with negative emotion symptomatology they may experience while going through treatment that could put them at greater risk for relapse.

Peck and colleagues (60) examined the temporal relationship between depressive symptoms and methamphetamine use among a sample of MUD gay and bisexual men undergoing 16 weeks of behavioral therapy. Patients were randomly assigned to one of four behavioral treatments: CBT, CM, CBT + CM, or gay-specific CBT. Approximately 28.5% of participants reported moderate to severe depression at the start of treatment, and all participants reported a decrease in depression by the end of treatment, regardless of condition. Additionally, patients reported reduced methamphetamine use up to one-year post-treatment. This suggests a strong connection between methamphetamine use and co-occurring depressive symptoms. However, methamphetamine use up to 5 days prior was found to predict depression ratings, but depressive symptoms were not found to predict methamphetamine use. This finding is interesting because it strengthens the hypothesis that depression may be a result of methamphetamine use rather than a motivating factor. The authors conclude that extended abstinence results in reduced depressive symptoms. However, methamphetamine use is likely reinforced by immediate relief of depressive and withdrawal symptoms, despite its long-term perpetuation of depressive symptoms (60).

Long-term stimulant use has been shown to modify how stress is processed, which can be detrimental to recovery from addiction (57). A pilot study investigated the use of MBRP in reducing stress-response among 22 adults with a stimulant use disorder. Patients were randomized to an 8-week intervention consisting of either MBRP or a health education program. Patients completed the Trier Social Stress Task pre- and post-intervention and provided self-report ratings of stress, anxiety, mood disturbance, and craving. Saliva samples were collected immediately following the stress task as well as 15, 30, and 60 min post-task as a measure of cortisol levels. Individuals in the MBRP group had significantly lower salivary cortisol levels 15 and 60 min after the stress task (29% and 24% variance explained, respectively). Additionally, MBRP patients had lower levels of subjective stress, anxiety, mood disturbance, and craving after the stress test administered post-treatment. This study shows promise for the use of MBRP to modify the way stimulant addicted individuals respond to stress; however, this study did not report on any substance use outcomes, so no conclusions can be drawn about whether MBRP is effective at reducing substance use. This study also did not differentiate between individuals addicted to cocaine versus methamphetamine. Although these drugs are both stimulants, they have different chemical properties, which research suggests may have differential effects (106). Regardless, this study suggests promise for the use of MBRP for treating substance use disorders by reducing stress, anxiety, and craving.

As demonstrated above, poor emotion/affect regulation can contribute to continued substance use. In addition to interventions attempting to decrease negative affect, there is also some promise for interventions attempting to enhance non-drug related positive affect. This was demonstrated in a sample of 110 MA sexual minority men positive for human immunodeficiency virus (HIV), who were randomly assigned to receive either CM combined with a positive affect (CM+PA) intervention or an attention-control condition (54). Patients in the CM+PA condition reported increases in positive affect (*d* = 0.31) and mindful awareness (*d* = 0.36) 3-months post-intervention, two factors related to emotion regulation. Importantly, these improved psychological processes were found to correspond with decreased craving (*d* = -0.51) and substance use *(d* = -0.46) at the 3-month follow-up (54). This finding suggests that positive affect interventions have the potential to improve substance use outcomes by: (1) increasing reward responsivity to non-drug related rewards; and (2) increase emotional processing in a way that reduces negative reinforcement. This supports the hypothesis that negative reinforcement plays an important role in the perpetuation of problematic substance use and that psychological interventions seeking to improve emotion regulation and stress response can simultaneously improve psychological factors as well as substance use outcomes.

### Exercise Interventions

Exercise has generally been shown to aid in the reduction of anxiety and depression (107), suggesting that it may be useful in reducing these uncomfortable sensations in methamphetamine addiction. A few studies have shown promising results for the use of exercise as either a primary or additive intervention for problematic methamphetamine use. In addition to treatment as usual, MUD newly enrolled in a residential treatment program for problematic methamphetamine use were randomly assigned to receive 8 weeks of either a health education control group or exercise program consisting of a 60-min structured exercise sessions three times per week. Among patients assigned to the exercise program, reductions in depression and anxiety symptoms were reported at the end and a dose effect on mood symptoms was also observed (63). Unfortunately, this study did not examine the relationship between depression and anxiety symptoms and treatment outcome variables related to substance use. However, other researchers have found that exercise, when compared to an attentional control group, reduced drug craving among MUD during and after a 12-week aerobic exercise program [$\eta_{p}^{2}$ = 0.16; Wang et al. (65)], and up to 50 min after an acute 30-min exercise session [$\eta_{p}^{2}$ = 0.34; Wang et al. (64)] . Together, these data suggest that reductions in anxiety and depression symptoms may mediate the relationship between exercise and reduced craving. Further studies are warranted to support this conclusion.

### Pharmacotherapy Interventions

Various medications have been investigated for the treatment of MUD. In line with the theory of negative reinforcement, it is hypothesized that antidepressant medications may improve substance use outcomes by treating mood symptoms that can precipitate relapse (108). Bupropion, sertraline, and mirtazapine are three antidepressant medications that have been examined within randomized, placebo-controlled trials. Two trials examined CM with either bupropion (70) or sertraline (69) in comparison to placebo. Outcome variables of interest included methamphetamine use, severity of depressive symptoms, and drug craving; however, no significant differences were found between either medication group and the placebo groups. These results suggest that bupropion and sertraline do not effectively reduce depressive symptoms among MUD above and beyond CM alone. Bupropion was also examined in conjunction with CBT. Again, no significant differences in craving or depressive symptoms were found between groups (bupropion vs. placebo), providing further evidence to suggest that bupropion is not effective for the treatment of MUD (67). A lack of group differences in reductions of depressive and anxiety symptoms have also been found following treatment with mirtazapine in conjunction with narrative therapy counseling compared to placebo (66). Overall, these trials imply that antidepressant medications do not reduce negative mood symptoms, and in turn, improve treatment outcomes in MUD beyond the effects of co-occurring interventions including CM, CBT, and narrative therapy. However, this evidence does not suggest that mood symptoms do not play a role in negatively reinforcing methamphetamine use; rather, it leaves the question of whether effectively treating mood symptoms can improve substance use outcomes unanswered.

In addition to antidepressant medications, other classes of drugs have been investigated for the treatment of MUD in conjunction with psychotherapy interventions. Previous research has suggested that GABAergic medications may be effective for the treatment of cocaine use, suggesting it may have similar efficacy for MUD (68). Thus, treatment-seeking MUD were randomized to receive either baclofen, gabapentin, or placebo in addition to attending relapse prevention groups. All three groups showed reductions in outcome measures including craving, retention, and depression scores over time with no significant difference between groups (68). The same research group investigated modafinil compared to placebo in conjunction with CM and CBT for MUD (70). This trial yielded similar results, wherein all patients reported reduced depressive symptoms regardless of medication condition. Additionally, there were no significant group differences for craving, methamphetamine use, or retention. Lastly, aripiprazole, an anti-psychotic, was investigated given its potential to increase dopamine transmission in light of reduced striatal dopamine levels among MUD (109, 110). However, similar to other trials investigating adjunctive medications for the treatment of MUD, aripiprazole did not appear to significantly reduce methamphetamine use, depressive symptoms, or craving beyond placebo (70). Further, individuals who received aripiprazole reported experiencing an increase in the rewarding and stimulatory effects after methamphetamine dosing, suggesting that this medication is unlikely to be efficacious for the treatment of MUD.

### Brain Stimulation Interventions

Based on fMRI findings of altered functioning in various frontal regions among MUD, repetitive transcranial magnetic stimulation (rTMS) delivered to these regions has been examined as a potential treatment intervention for addiction. rTMS delivers noninvasive stimulation to specific cortical regions by applying a fluctuating magnetic field between 0-10 Hz [Liang et al. (71)]. As frontal processing deficits may contribute to difficulties with attention and emotion regulation, resulting in an inability to adjust behavioral responses accordingly (44), using rTMS to alter neural activity in frontal regions may result in improved emotional functioning. Although the literature is limited, two studies found that, in comparison to sham control groups, 10 Hz rTMS delivered to left DLPFC decreased depression and anxiety symptoms in men with MUD (71, 72). Observed reductions in symptoms of anxiety also related to reductions in MA craving (71). An additional study reported that real rTMS reduced craving, but both real and sham rTMS decreased depressive symptoms, while neither condition resulted in any change in anxiety symptoms (111). These contradictory findings may be due to differences in study design as reductions in depressive symptoms were found after 10 or 30 rTMS sessions, but not after five sessions, suggesting that change in mood symptoms may be dose dependent. Currently, there is no research examining rTMS in female MUD patients, nor longitudinally to determine if any effect on mood symptoms is sustained over time. Further research is needed to elucidate the effects of rTMS on mood symptoms.

In line with the theory that methamphetamine use is negatively reinforced by the relief of negative mood states, treating symptoms of anxiety and depression holds promise for improving substance use outcomes. Findings are mixed with regard to efficacy of psychotherapy, exercise, pharmacotherapy interventions, and brain stimulation. Various psychotherapy treatments including CBT, CM, MBRP, MI, and positive affective interventions have shown promise for reducing mood symptoms and thereby improving substance-related treatment outcomes including greater treatment adherence, and reduced craving and methamphetamine use (see **Table 3**). Exercise may also improve treatment outcomes among MUD by reducing anxiety and depressive symptoms as well as craving. rTMS may also hold promise for improving mood symptoms and reducing craving but the research is too limited at this time to draw any strong conclusions. Less compelling evidence has been found for the use of adjunctive medications in the treatment of MUD. Multiple controlled-trials have been unable to demonstrate any significant reductions in outcomes related to mood symptoms or substance use above and beyond placebo. Regardless, some progress has been made in the treatment of MUD, but further research is warranted to improve treatment outcomes. Targeting negative mood symptoms related to anxiety and depression appears to be a promising avenue for effectively improving treatment outcomes among MUD.

## Limitations and Future Directions

The findings related to negative reinforcement in MUD suggest a number of promising avenues for future treatment research. One such avenue is emotion-focused interventions. One study demonstrated a positive affect intervention to be effective at improving emotion regulation processes, thereby reducing drug craving and use among HIV-positive sexual minority men with MUD (54). Given these promising findings within a specific subpopulation of individuals with MUD, additional research is warranted to examine the efficacy of emotion-focused interventions in the general MUD population. Additionally, considerable efforts should be put towards developing emotion-focused interventions that specifically target suicidal ideation given the high rates of suicide among MUD. Based on the findings outlined above, interventions aimed at helping individuals develop efficient emotion regulation and healthy coping skills hold promise to effectively reduce emotional symptoms common among MUD and in turn improve substance use outcomes. Additionally, while some improvement has been found with other interventions (i.e., CBT, CM, MI, and MBRP), features of anxiety and depression, such as severity, have been found to predict poorer adherence to such treatments (55). This further highlights the need for emotion-focused interventions that target negative mood symptoms that maintain and exacerbate substance use disorders. Adjunctive pharmacotherapy may also prove effective in reducing mood symptoms to allow for better treatment adherence, although the evidence is less compelling.

Efforts should be made to develop and test interventions that alter activity in brain regions in which MRI, PET, and ERP research have demonstrated deficits among MUD. Current findings among MUD suggest that brain stimulation may be one intervention effective in modifying brain activity. rTMS of DLPFC has been applied to MUD with mixed results in terms of changes in mood symptoms and drug craving (71, 72, 111). Additional research should examine if these changes in mood and craving coincide with sustained abstinence/reductions in use and whether rTMS can effectively increase executive functioning and enable MUD to choose adaptive behavioral responses despite negative emotional symptoms. rTMS has yet to be applied to brain regions other than DLPFC that exhibit altered functioning among individuals with MUD.

Various other interventions that have been shown to modify brain function in non-substance-using individuals may be potential treatments for targeting brain regions altered in MUD. These include mindfulness meditation [e.g., Taren et al. (112)], behavioral activation therapy [e.g., Dichter et al. (113)], and trauma-focused therapy [e.g., Aupperle et al. (114), Simmons et al. (115)], have been found to impact brain function in circuitry considered important for emotional processing and regulation and have beneficial effects for negative affect related symptoms. Using related strategies with MUD populations (or particularly those with co-occurring depression, anxiety, or PTSD) may therefore be beneficial for interrupting the negative reinforcement cycle. Additionally, other pharmacological interventions may also be useful for altering dysfunctional brain regions in MUD, such as modafinil, which has been shown to increase insula and ACC RSFC with other brain regions (116). By exploring interventions that target dysfunctional brain regions highlighted in the literature on MUD, researchers may be able to develop treatments that break the negatively reinforcing cycle of using methamphetamine to reduce uncomfortable sensations.

Overall, our understanding of negative reinforcement in MUD and its implications for treatment is hindered by limitations in the research. In addition to the potential avenues of treatment research outlined above, future researchers should aim to address the following limitations. First, many findings come from studies of small sample sizes and specific populations (e.g., HIV-positive, sexual minorities) which limits the ability to generalize to the MUD population overall (see **Tables 2** and **3**). Second, the prevalence of cross-sectional studies greatly limits the inferences that can be made regarding causation of observed individual differences (i.e., emotional processing deficits). Longitudinal studies would allow for examination of the temporal relationship between emotion dysregulation and MUD and the results could potentially inform the development of successful prevention efforts. For example, the Adolescent Brain Cognitive Development (ABCD) study began in 2016 and is the largest long-term study of brain development to date, following a cohort of approximately 11,500 youth for ten years. The data from the ABCD study hold promise for elucidating the relationship between emotion dysregulation and substance use disorders as it will allow for a prospective examination of these problems as they develop. Longitudinal treatment studies would also be useful to determine whether the observed deficits observed in long-term methamphetamine users are predictive of relapse and other treatment-related outcomes. Third, reported data on drug use characteristics such as duration of use, recency of use, duration of abstinence, etc., is varied and lacking. This information is crucial to examine in relation to observed behavior and brain functioning to better understand the interaction between substance use and unfavorable outcomes. This could also aid our understanding of which interventions are most effective and for whom. Lastly, there is a lack of treatment studies coupled with neuroimaging. Pairing these methods together would allow researchers to determine whether an intervention impacts brain networks that are dysfunctional in MUD (e.g., executive function, reward processing, and emotion regulation) and whether it is likely to impart lasting change.

The current body of literature appears to preliminarily support the hypothesis that negative reinforcement is at play in the development and maintenance of MUD. However, the majority of the studies included in this review employed cross-sectional and/or quasi-experimental designs, which do not allow for the precise testing of the longitudinal nature of the three-stage model of addiction (9). There is room for continued research efforts to further clarify the extent to which negative reinforcement contributes to substance use disorders and whether interrupting these processes holds value as a potential treatment option.

## Author Contributions

AM contributed to the development of the concept for the review, completed literature search, and wrote the first draft of the manuscript. JS and RA both contributed to the development of the concept for the review, and revised subsequent drafts of the review.

## Funding

This work was supported by The William K. Warren Foundation and the National Institute on Alcohol Abuse and Alcoholism (F31AA027169).

## Conflict of Interest

The authors declare that the research was conducted in the absence of any commercial or financial relationships that could be construed as a potential conflict of interest.
